# CT Marker in Emergency Imaging of Acute Basilar Artery Occlusion: Thrombosis vs. Embolism

**DOI:** 10.3390/diagnostics12081817

**Published:** 2022-07-28

**Authors:** Fabian Mueck, Moritz Hernandez Petzsche, Tobias Boeckh-Behrens, Christian Maegerlein, Ulrich Linsenmaier, Mariano Scaglione, Claus Zimmer, Benno Ikenberg, Maria Berndt

**Affiliations:** 1 Institute for Diagnostic and Interventional Radiology, HELIOS Clinics Munich West, Munich Perlach & Augustinum Munich, 81241 Munich, Germany; fabian.mueck@helios-gesundheit.de (F.M.); ulrich.linsenmaier@helios-gesundheit.de (U.L.); 2 Department of Diagnostic and Interventional Neuroradiology, Klinikum Rechts der Isar, School of Medicine, Technical University of Munich, 81675 Munich, Germany; moritz.hernandez@tum.de (M.H.P.); boeckh-behrens@tum.de (T.B.-B.); christian.maegerlein@tum.de (C.M.); claus.zimmer@tum.de (C.Z.); 3 Department of Radiology, University of Sassari, 07100 Sassari SS, IT and James Cook University Hospital, Middlesbrough TS4 3BW, UK; mscaglione@uniss.it; 4 Department of Neurology, Klinikum Rechts der Isar, School of Medicine, Technical University of Munich, 81675 Munich, Germany; benno.ikenberg@mri.tum.de

**Keywords:** stroke, basilar artery occlusion, CT-angiography, perviousness, emergency, thrombus, embolism, outcome, stenosis

## Abstract

**Purpose:** Acute basilar artery occlusion, a neurovascular emergency leading to high rates of morbidity and mortality, is usually diagnosed by CT imaging. The outcome is partly dependent on etiology, with a worse outcome in occlusions with underlying basilar artery stenosis. As this occlusion type requires a more complex angiographic therapy, this study aimed to develop new CT markers in emergency admission imaging to rapidly identify underlying stenosis. **Methods:** A total of 213 consecutive patients (female n = 91, age in years (mean/SD/range): 72/13/28–97), who received endovascular treatment at a single comprehensive stroke center for acute basilar artery occlusion, were included in this study. After applying strict inclusion criteria for imaging analyses, novel CT imaging markers, such as ‘absolute density loss’ (ADL) and relative thrombus attenuation (CTA-index), that measure perviousness, were assessed for n = 109 patients by use of CT-angiography and correlated to different occlusion patterns (thrombotic vs. embolic). Inter-observer agreement was assessed using an intraclass correlation coefficient for independent measures of a radiologist and a neuroradiologist. Associations between the imaging markers and clinical and interventional parameters were tested. **Results:** CT markers differ between the subgroups of basilar artery occlusions with and without underlying stenosis (for ADL: 169 vs. 227 HU (*p* = 0.03), for CTA-index: 0.55 vs. 0.70 (*p* < 0.001)), indicating a higher perviousness in the case of stenosis. A good inter-rater agreement was observed for ADL and CTA-index measures (ICC 0.92/0.88). For the case of embolic occlusions, a more pervious thrombus correlates to shorter time intervals, longer procedure times, and worse reperfusion success (*p*-values < 0.05, respectively). **Conclusions:** ADL and CTA-index are easy to assess in the emergency setting of acute basilar artery occlusion with the use of routinely acquired CT-angiography. They show a high potential to differentiate thrombotic from embolic occlusions, with an impact on therapeutic decisions and angiographic procedures. Measurements can be quickly performed with good reliability, facilitating implementation in clinical practice.

## 1. Introduction

Acute basilar artery occlusion (BAO) is a rare form of stroke (about 1%) resulting in high rates of disability and mortality, especially without revascularization therapy [1,2,3]. Despite limited level I evidence [4,5,6], the standard therapy for acute basilar artery occlusion in most stroke centers is mechanical thrombectomy (MT), with high reperfusion rates and improvements in clinical outcome comparable to the anterior circulation [7,8,9]. We live in the era of the proven effectiveness of MT in the anterior circulation, and the majority of observational studies also show a clear benefit of MT in posterior circulation strokes [10,11]. Data from large national or international multicenter registries [12] further support the benefits of MT in BAO.

Acute BAO is known to present with variable clinical symptoms, with high inter-individual variability. Hereby, pathophysiological mechanisms may play an important role, especially in the etiology of the thrombo-embolic event.

In comparison to acute occlusions of the middle cerebral artery, there is a higher rate of underlying local atherosclerosis, resulting in basilar artery stenosis (BS), followed by a local thrombosis as a cause of BAO. For this entity of in situ atherosclerotic thrombosis, lower rates of successful recanalization and worse clinical outcome are reported [13,14].

Most of the BAO cases with underlying BS are treated with MT, followed by angioplasty and permanent stent placement, which warrants a more complex angiographic procedure [15]. As reported previously [16], it might be helpful to identify the thrombotic cases with underlying BS before starting the endovascular procedure. The imaging marker perviousness showed a high potential to differentiate acute BAO with and without BS [16]. However, the assessment of thrombus perviousness is technically demanding and warrants accurate measurements that are not implementable in the acute emergency setting of each radiological department without fully workflow-integrated software.

Diagnosis of an acute BAO is usually made by CT imaging, including CT-angiography. The present study aimed to develop new CT imaging markers that should be easily assessable in an emergency setting by the radiologist, to rapidly identify a thrombotic BAO with underlying BS.

## 2. Material and Methods

As the primary endpoint, imaging markers were assessed by admission CT-angiography (CTA) within the primary cohort of patients with acute occlusion of the basilar artery (labeled “perviousness cohort”). Analogous to previous perviousness measures, the acquired imaging parameters were tested for their capacity to identify the underlying stenosis of the acute BAO.

As a secondary endpoint, associations were tested in a second independent group of patients with acute BAO for validation purposes by two independent readers (labeled “validation cohort”).

Ethical approval was obtained from the local ethics committee. Informed consent for this study was waived due to the retrospective nature of this work.

### 2.1. Sample and Patient Description

This retrospective, single-center study included all consecutive patients with ischemic stroke due to an acute occlusion of the basilar artery who were admitted at a single comprehensive stroke center between March 2008 and March 2022 (n = 213). In all patients, thrombo-embolic material was visualized within the basilar artery and verified by CT- or MR-angiography. The whole cohort was divided into two independent subgroups:

The perviousness cohort (n = 115) consisted of a homogenous collective of patients with acute BAO, consecutively treated with second-generation thrombectomy devices between March 2008 and August 2017 (already described in [16,17]).

The independent validation cohort (n = 98) consisted of an analogous collective of patients with an acute BAO, who were consecutively treated between September 2017 and March 2022.

**Figure 1:**
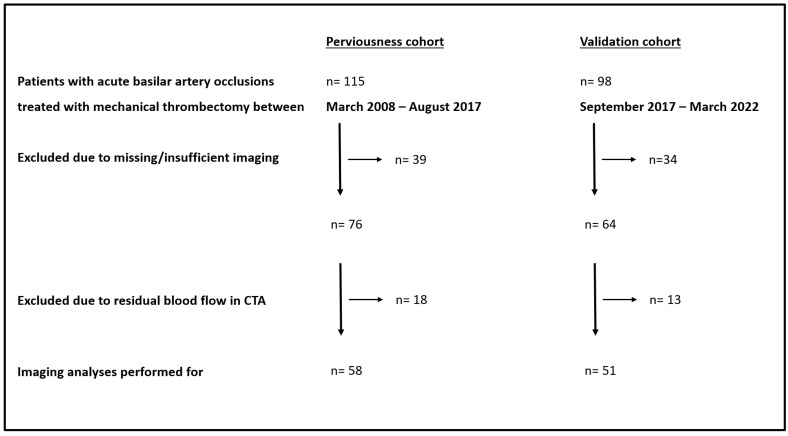
Study flow chart: exact numbers and reasons for exclusion in the perviousness and validation cohort. CTA indicates computed tomographic angiography.

Inclusion criteria for the assessment of imaging markers in the admission imaging were sufficient pre-interventional imaging (CTA) with a thickness <3 mm and a complete occlusion without proof of residual blood flow. The study flow chart with exact numbers and reasons for exclusion for both cohorts can be found in Figure 1.

The prospectively-collected clinical and imaging data were retrospectively analyzed (BI, >10 years of neurological experience). Basic demographic, clinical, and interventional data of patients were gathered. NIHSS-certified neurologists assessed the National Institutes of Health Stroke Scale (NIHSS-) score at the time of admission and discharge. Substantial neurological improvement was defined as the difference between admission and discharge NIHSS score of ≥8, or discharge NIHSS score of ≤ 1, as described previously [17,18,19]. MRS-score was used to measure disability at discharge, while a good clinical outcome at discharge and in the 3-month follow-up was defined as mRS ≤ 3 for the posterior circulation.

Successful recanalization was defined as mTICI 2b-3, complete recanalization as mTICI 3. Time of symptom onset, time of groin puncture, time of reperfusion, and corresponding procedure times were taken from the existing database. The time interval was defined between symptom onset and groin puncture. Procedure time was defined as the time difference between groin puncture and reperfusion. In cases when recanalization was not successful (mTICI < 2b), the control series after the last maneuver was used as the time endpoint. Further treatment variables that are relevant for the present study included administration of pre-interventional intravenous tissue-type plasminogen activator (tPA), the number of maneuvers during mechanical thrombectomy, and the necessity of additional stenting of remaining stenosis.

Stroke pathogeneses were determined according to the international Trial of ORG 10,172 in Acute Stroke Treatment (TOAST) classification [20] based on the diagnostic and clinical information available for each patient. BAO rated as TOAST 1 (large-artery atherosclerosis) could be based on either embolism (e.g., due to vertebral stenosis with plaques) or local thrombosis due to underlying stenosis. This was identified on interventional digital subtraction angiography images by visualizing vessel wall abnormalities leading to stenosis of the basilar lumen (in the consensus reading of two experienced neuroradiologists). In three cases, classification was not possible due to the failure of vessel reperfusion.

### 2.2. Imaging Analysis

For all included patients, imaging markers were assessed using admission CT imaging.

#### 2.2.1. Admission Imaging Technique

For all included patients, admission imaging, including unenhanced cranial CT and CTA, was performed before endovascular therapy.

In our department, computed tomography angiography (CTA) was performed as a helical scan, with coverage from vertex to aortic arch and continuous axial sections parallel to the orbitomeatal line with 0.6 mm section thickness. CTA was performed either on a 64-row (CT 1: Philips Brilliance 64, Philips Medical Systems B.V., AE Eindhoven, Best, The Netherlands) or 128-row CT scanner (CT2: Philips Inguinity 128, Philips Medical Systems B.V., AE Eindhoven, Best, The Netherlands).

A dual-head power injector (Medrad, Warrendale, PA, USA) with an 18-G i.v. access was used for contrast injection. A bolus of 70 mL Iomeron^®^ (Bracco, Milan, Italy) at a flow rate of 4 mL/s was applied, followed by 50 mL NaCl. The scan was auto-triggered by the appearance of contrast in a region of interest (ROI) manually placed in the ascending aorta (attenuation > 150 HU).

The following parameters were used, varying due to dose modulation:

CT 1: 120 kV, 150–400 mA dose modulation, 0,5 s Rotation time, pitch 1.015

CT 2: 120 kV, 100–350 mA dose modulation. 0,5 s Rotation time, pitch 1.015

#### 2.2.2. Assessment of Imaging Markers

**Figure 2:**
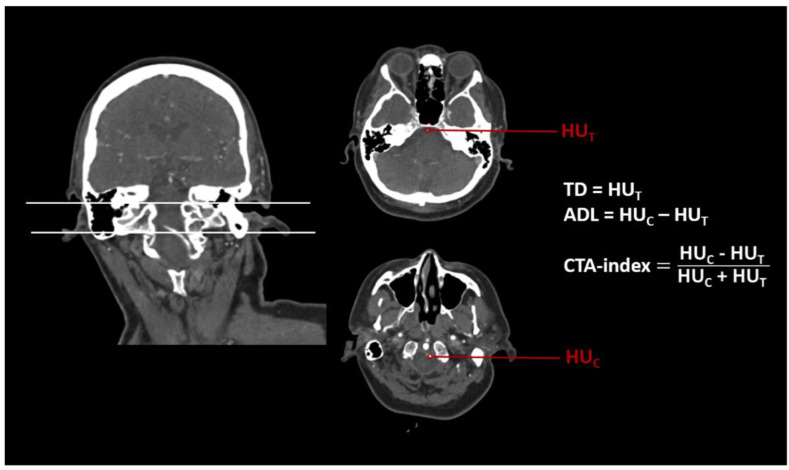
Example of an assessment of CT imaging markers pure thrombus density (TD), absolute density loss (ADL), and CTA-index (relative thrombus attenuation) for a patient (male, 60 years) with an acute basilar artery occlusion. ROIs for measurements of mean Hounsfield Units (HU) were placed at the occlusion site (T) and a reference point (C).

Within the perviousness cohort, thrombus perviousness parameters were already assessed (see [16]). Mean density values of the CTA in ROIs (a) within the occlusion site (labeled as (T), measuring points were placed about 1.5 mm distal of the visible stop of contrast agent in consideration of appositional thrombus growth) and (b), at a reference point (labeled as (C), within a preceding vessel section that is regularly filled with contrast agent at a distance to the occlusion site), were used for further analyses. Imaging markers such as pure thrombus density in CTA (TD = HU_T_), absolute density loss (ADL = HU_C_ − HU_T_), and relative thrombus attenuation (CTA-index = (HU_C_ − HU_T_)/(HU_T_ + HU_C_), modified according to [21]), were calculated for each patient (Figure 2).

The same imaging markers were assessed for all patients of the validation cohort, applying the same inclusion criteria.

The measurements were applied by a neuroradiologist (>4 year of neuroradiological experience, MB). To test for inter-observer agreement, a senior radiologist (>10 year of radiological experience, FM), blinded to all clinical data and the analysis performed, repeated the measurements of imaging markers within the validation cohort.

### 2.3. Statistical Analysis

In an explorative approach, various variables were tested for an association with imaging markers. Two-sample *t*-tests were used for parametric variables, Wilcoxon rank-sum tests for nonparametric variables, and Fisher exact test for dichotomous categorical variables.

Intraclass correlation coefficient (ICC) was used to assess the agreement of imaging examinations (for ADL and CTA-index), rated by two independent neuroradiologists (FM, MB), using the two-way mixed-effects model [22]. Receiver operating characteristic (ROC) analyses were implemented to assess the accuracy of the imaging markers to discriminate BAO with and without BS. Thresholds were identified beyond which the imaging markers ADL and CTA-index, respectively, might show reasonable discriminatory power to indicate underlying BS, and calculations of the Youden Index were added.

All statistical analyses were performed using IBM SPSS Statistics (version 28, IBM Corp, Armonk, NY, USA).

## 3. Results

### 3.1. Patient Characteristics

The study cohort consisted of 213 patients with ischemic stroke due to acute BAO, who were consecutively endovascular treated with MT at a single stroke-center between March 2008 and March 2022.

An overview of demographic, clinical, and interventional data of patients within the two subgroups can be found in Table 1.

### 3.2. Introducing Imaging Markers as a Simplified Assessment of Perviousness

From preceding perviousness measures, density values of CTA image ROIs within the thrombus and the reference point of the perfused vessel were extracted (Figure 2). ADL and CTA-index were assumed to measure perviousness. Lower values indicated a higher perviousness.

**Figure 3:**
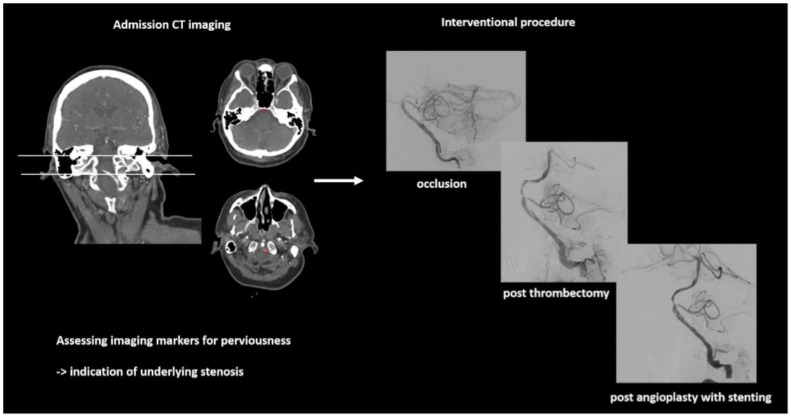
Example of a patient (male, 60 years) with acute occlusion of the basilar artery. In admission imaging, CT markers for perviousness were assessed. During endovascular therapy, underlying stenosis was detected after the first mechanical thrombectomy, resulting in recurring thrombus formation and the necessity for further thrombectomy maneuvers and final angioplasty with permanent stent placement.

Within the perviousness cohort (n = 58 met the criteria for imaging analyses) and according to previous perviousness analyses, the group of BS (Figure 3) was analyzed in respect of imaging markers in comparison to the group of BAO without BS. There were no differences for TD (BS/no BS: median (IQR) in HU: 66 (61–95)/68 (55–96), *p* = 0.82). The group of BS had lower values for ADL (in HU: 91(81–150) vs. 173 (125–234), *p* = 0.002) and lower values for CTA-index (0.40 (0.39–0.54) vs. 0.59 (0.46–0.70), *p* = 0.004).

### 3.3. Validation of Analyses in a Second Cohort: Capacity of Identifying Underlying Stenosis

In the validation cohort, 51 patients met the criteria for imaging analyses. The median TD was 59HU (IQR 48–83), median ADL was 205HU (IQR 155–253) and median CTA-index was 0.66 (IQR 0.55–0.74).

**Figure 4:**
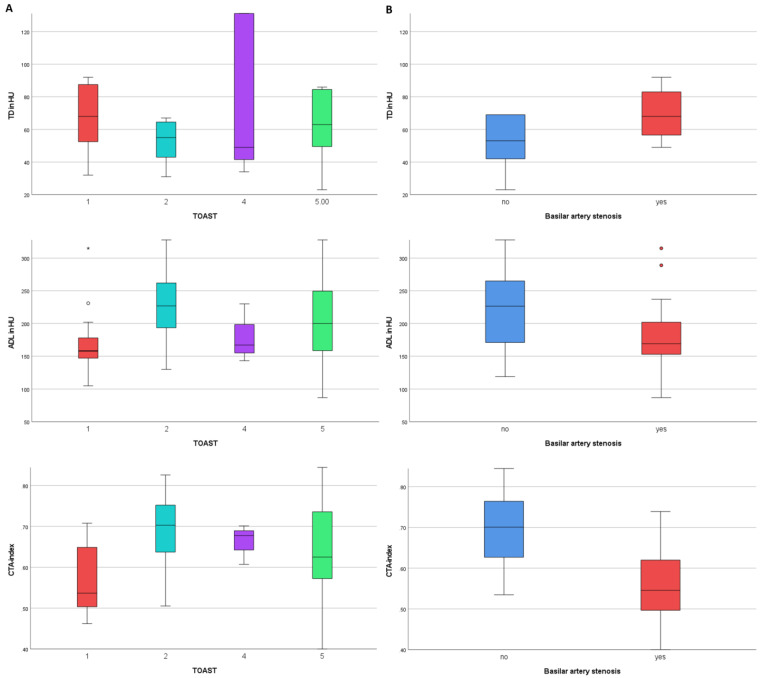
Distribution of the measured imaging markers for the different etiological groups (according to TOAST) in (**A**) and for the presence of underlying basilar artery stenosis in (**B**), with significant differences for values of imaging markers between the groups of BS vs. no BS, respectively. TD: pure thrombus density in CTA, ADL: absolute density loss.

The distribution of the measured imaging markers for the different etiological groups can be found in Figure 4A. Imaging markers were compared for the groups of BAO with and without BS (Figure 4B): There was a difference for TD (BS/no BS: median (IQR) in HU: 68 (57–83)/53 (42–69), *p* = 0.04). The group of BS had lower values for ADL (in HU: 169 (153–202) vs. 227 (171–265), *p* = 0.03) and lower values for CTA-index (0.55 (0.50–0.62) vs. 0.70 (0.63–0.76), *p* < 0.001).

**Figure 5:**
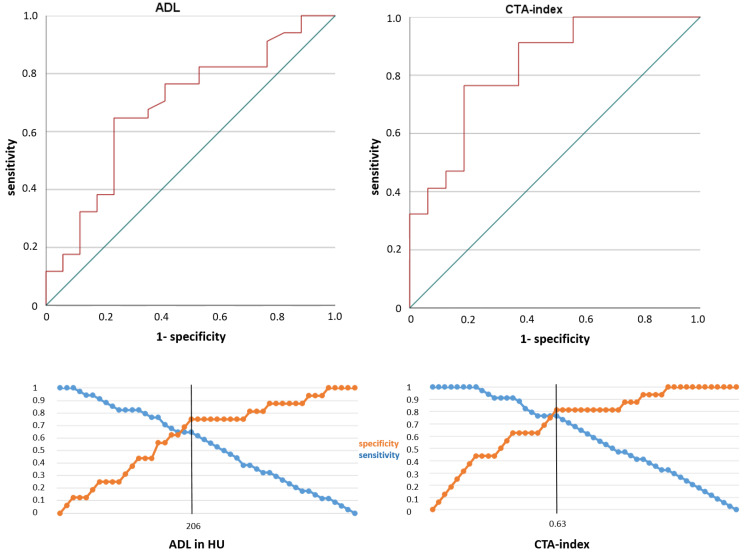
Analyses to identify underlying basilar artery stenosis in acute basilar artery occlusions. Receiver operating characteristics (top) and sensitivity/specificity (bottom) curves of absolute density loss (ADL) and relative thrombus attenuation (CTA-index).

ROC analyses for the imaging markers ADL and CTA-index indicated that these parameters are significant indicators of the presence of no BS (vs. BS), with the area under the curve (AUC) of 0.69 [95%-CI: 0.53–0.84, *p* = 0.03, accuracy ratio 0.38] for ADL and AUC of 0.83 [95%-CI: 0.70–0.95, *p* < 0.001, accuracy ratio 0.66] for CTA-index, both implicating good models (Figure 5). According to the ROC analysis, a specificity of over 80% to identify a BAO without BS in admission CT imaging would be reached for a cutoff value of >206 HU for ADL (Youden index 0.40, specificity 75%, at a sensitivity of 65%) and a cutoff value of >0.63 for CTA-index (Youden index 0.58, specificity 81%, at a sensitivity of 77%).

### 3.4. Reliability of CT Imaging Markers

The inter-rater reliability of the measurements between the radiologist and the neuroradiologist was tested. A good inter-rater agreement was observed for ADL (ICC of 0.92 (CI 0.85–0.95)) and the CTA-index (ICC of 0.88 (CI 0.79–0.93). This suggests good reliability of the imaging markers within the study´s setting with observer independency.

### 3.5. Association to Clinical and Interventional Parameter

For the whole cohort with imaging analyses (n = 109), values of ADL and CTA-index poorly correlated with time interval (for ADL/CTA-index: r = 0.24/0.23, *p* = 0.03/0.04) and negatively with procedure time (for ADL/CTA-index: r = −0.29/−0.37, *p* = 0.002/<0.001). Only a weak association was seen with the number of maneuvers (for ADL/CTA-index: r = −0.16/−0.16, *p* = 0.11/0.10). There was a poor correlation between ADL/CTA-index and reperfusion success (mTICI, r = 0.23/0.22, *p* = 0.02/0.02).

The same analyses were repeated after excluding the cases of underlying basilar artery stenosis, as they usually result in a more complex angiographic procedure. Correlations were also seen between ADL/CTA-index and time interval (r = 0.47/0.44, *p* = <0.001/<0.001), and procedure time (r = −0.26/−0.36, *p* = 0.02/0.002). a weak association was seen with number of maneuvers (for ADL/CTA-index: r = −0.08/−0.13, *p* = 0.47/0.25). ADL/CTA-index values correlate poorly with reperfusion success (mTICI, r = 0.23/0.17, *p* = 0.04/0.15).

No associations were found between imaging markers and clinical values at admission, at discharge, and the 3-month follow-up (*p* > 0.05). As is well known, BAO with underlying BS presents with worse clinical outcomes (good clinical outcome for the whole cohort (BS vs. no BS): n = 18% vs. 45%, *p* < 0.001). Analyzing the subgroup of BAO without underlying BS, no associations were found between imaging markers and clinical values (*p* > 0.05). Analyzing the subgroup of BAO with underlying BS, a negative correlation was found between ADL/CTA-index and mRS at 3-month follow-up (r = −0.55/−0.66, *p* = 0.03/0.01), indicating that a more pervious thrombus would show a worse clinical outcome.

## 4. Discussion

The present study introduces novel CT markers in the emergency admission imaging of acute BAO, as demonstrated in a large single-center cohort. The parameters ADL and CTA-index, as simplified measures for perviousness, can be easily and quickly assessed in the admission CT and do not warrant prior specialized neuroradiological experience. Good inter-rater reliability indicates robustness and facilitates the future implementation of these techniques into clinical routine.

Similar to known perviousness measurements [16], these simplified CT markers can be applied to differentiate BAO due to BS from embolic BAO.

The perviousness cohort was used to screen the new parameters. The results suggested that ADL and CTA-index could replace the conventional perviousness measures in their capacity to detect underlying stenosis. Validation in a second, independent cohort of patients with acute BAO was performed independently by a radiologist and a neuroradiologist testing the setting of an emergency. ADL and CTA-index measurements in the validation cohort confirm their capacity to detect underlying stenosis.

The parameter of pure thrombus density in CTA (TD), assessed by placing the ROI within the occlusion site of the CTA image and part of the measurements of ADL/CTA-index, also showed the potential to differentiate BAO with and without underlying stenosis. However, solitary use of the TD parameter is not recommended as the robustness of this marker is limited. ADL and CTA-index contain a correction for the influence of circulation and contrast agent flooding, making them more accurate measurements of perviousness.

The assessment of perviousness for thrombus characterization of an acute large-vessel occlusion has been described several times. In the anterior circulation, perviousness measurements have been shown to provide information about stroke pathogenesis, thrombus histology, reperfusion success, and outcome prediction [23,24,25]. Due to differences in pathophysiology, including higher rates of an atherosclerosis resulting in BS, the results of the anterior circulation cannot simply be transferred to the posterior circulation [26,27]. With underlying BS plaque, rupture is followed by a local thrombus formation leading to acute BAO. When assessing perviousness for this local-thrombotic entity of BAO, measures are placed at the occlusion site that may include atherosclerotic plaque and appositional thrombus material, which are difficult to differentiate in CTA images. As reported earlier, unstable plaque formations may be visualized in CT by contrast enhancement due to neovascularization and plaque inflammation [28,29,30,31,32,33]. Perviousness may be a measure of plaque enhancement, explaining its sensitivity to detecting underlying stenosis [16]. As opposed to classical perviousness measurements, the unenhanced density values of the thrombus/plaque at the occlusion site are not taken into account. We show that it is not necessary to correct the approximated perviousness measures for the unenhanced density values to detect underlying stenosis, making prior co-registration in patients with head movement between scans redundant and facilitating applicability.

ADL is rapidly measured by placing ROIs within the identified occlusion site and within the upstream, still perfused vascular section, followed by subtraction of the measured Hounsfield Units. This measurement can be expected of any radiologist who is familiar with the broadly used CT-angiography in the acute setting of stroke imaging. Adding a simple, quick calculation, by inserting the two HU-values in a formula, the second parameter CTA-index (relative thrombus attenuation), can be generated, which is modified from the previously described parameter that was used as a simplified perviousness measure and proven for occlusions of the anterior circulation [21]. Compared to ADL, the CTA-index is more sophisticated, with better sensitivity and specificity. However, ADL may be more suitable for clinical routine, as it does not require a secondary calculation step, which may be a hindrance in an emergency setting.

Cut-off values for ADL and CTA-index, assessed by ROC-analyses, were generated to detect the underlying stenosis of an acute BAO in admission imaging. This local thrombotic entity of an acute BAO is known to present with lower rates of successful reperfusion and worse clinical outcome [13,14]. Identifying the underlying stenosis of acute BAO in the admission imaging before therapy initiation may have an impact on the further workflow. BAOs with underlying BS are usually treated with thrombectomy, followed by angioplasty with permanent stent placement. The diagnosis of underlying stenosis in BAO at admission may help in procedure preparation. In the case of BAO due to BS, the interventional team, including the anesthesiologist, can prepare for a longer procedure time and the possibility of stenting (including the preparation of suitable material) with subsequent anticoagulation. Furthermore, it may be used for outcome prediction, as acute BAO with BS is known to present with worse clinical outcomes. Additionally, in the case of BAO with underlying stenosis, perviousness measures were shown to correlate with 3-month follow-up mRS values. A more pervious occlusion site was associated with worse clinical outcomes, possibly due to a higher plaque instability of the underlying stenosis [28,29,30,31,32,33].

In the case of embolic origin of acute BAO without underlying stenosis, associations with different interventional parameters were shown. A more pervious thrombus correlates with a shorter time interval, a longer procedure time with a tendentially higher number of required maneuvers, and worse reperfusion success. This is in line with known associations between thrombus perviousness, histology, and etiology [25], as higher perviousness is related to fibrin-rich, cardioembolic clots. These associations could be transferred from the anterior to the posterior circulation strokes when only considering the embolic cases of the posterior circulation.

Besides its retrospective design, there are some further limitations of the study. The measurement of perviousness is not possible for calcified thrombi or plaques. Further analyses are required to find ways of dealing with calcified thrombi. A further limitation of the study is the risk of possible underestimation of perviousness due to timing limitations, hemodynamic restriction, or pseudo-occlusion that cannot be avoided in a single-phase CTA. Direct contact between the contrast agent and the thrombus is a prerequisite for perviousness assessment.

## 5. Conclusions

With ADL and CTA-index, novel CT imaging markers are introduced as simplified measurements of perviousness for acute basilar artery occlusions. They are easy to assess in emergencies regarding an acute basilar artery occlusion with the use of routinely acquired CT-angiography in admission imaging. The study’s conception showed the applicability of neuroradiologists in daily clinical practice. Perviousness measurements obtained by these CT markers may be used to differentiate thrombotic vs. embolic occlusions, with an impact on further therapeutic decisions and outcome prediction.

## Figures and Tables

**Table 1:** Patient characteristics. Baseline demographic, clinical, and interventional data for the perviousness and validation cohort. Results of univariate comparisons between the two groups are displayed.

Characteristics	Perviousness Cohort (n = 115)	Validation Cohort (n = 98)	Group Comparison (*p*-Value)
Age, y, median (IQR)	74 (63–81)	75 (63–82)	0.63
Sex, n (female/male)	39/76	52/46	<0.01
Additional intravenous thrombolysis (n (%))	53 (46%)	31 (32%)	0.03
Number of maneuvers (median/IQR)	2 (1–3)	1 (1–2)	<0.01
mTICI-Score post recanalization (n in%)			0–2a vs. 2b–3: *p* = 0.34
0–2a	7%	11%	
2b	26%	20%	
3	67%	69%	
Basilar artery stenosis (n/%)	31 of 115 (27%)	26 of 95 (27%)	0.9
Stenting in case of basilar artery stenosis (n/%)	26 (84%)	21 (81%)	0.9
Time interval (onset to the groin in min, median/IQR)	240 (190–315) for n = 81	288 (192–465) for n = 68	0.03
Procedure time (min, median/IQR)	69 (45–120)	41 (24–83)	<0.01
NIHSS (median, IQR)			
Pre-treatment (at admission)	15 (10–22)	10 (5–21)	0.03
Post-treatment (at discharge)	11 (3–38)	7.5 (2–42)	0.49
mRS-Score (90 days), n	n = 115	n = 90	
0–3 (good clinical outcome)	42 (36.5%)	34 (37.8%)	0.9
>3	73 (63.5%)	56 (62.2%)	

## Data Availability

The data analysis was based on anonymised data from the picture archive system (PACS), radiology information system (RIS) and hospital information system (HIS) at Department of Diagnostic and Interventional Neuroradiology, Klinikum Rechts der Isar, School of Medicine, Technical University of Munich. No publicly archived datasets were analyzed or generated during the study.

## Abbreviations

ADL absolute density loss; BAO basilar artery occlusion; BS basilar artery stenosis; CT Computed Tomography; CTA Computed Tomography Angiography; HU Hounsfield Units; ICC Intra-Class-Coefficient; mRS modified Rankin Scale; NIHSS National Institutes of Health Stroke Scale; mTICI modified thrombolysis in cerebral infarction; ROC Receiver operating characteristic; ROI Region-of-Interest; TD pure thrombus density in CTA.
